# Supplementary figures and images for: A pyroptosis-related gene signature for the diagnosis of acute pancreatitis
Source: PLoS One. 2026 Apr 17;21(4):e0347204. doi: 10.1371/journal.pone.0347204 (PMC13089757; doi:10.1371/journal.pone.0347204)

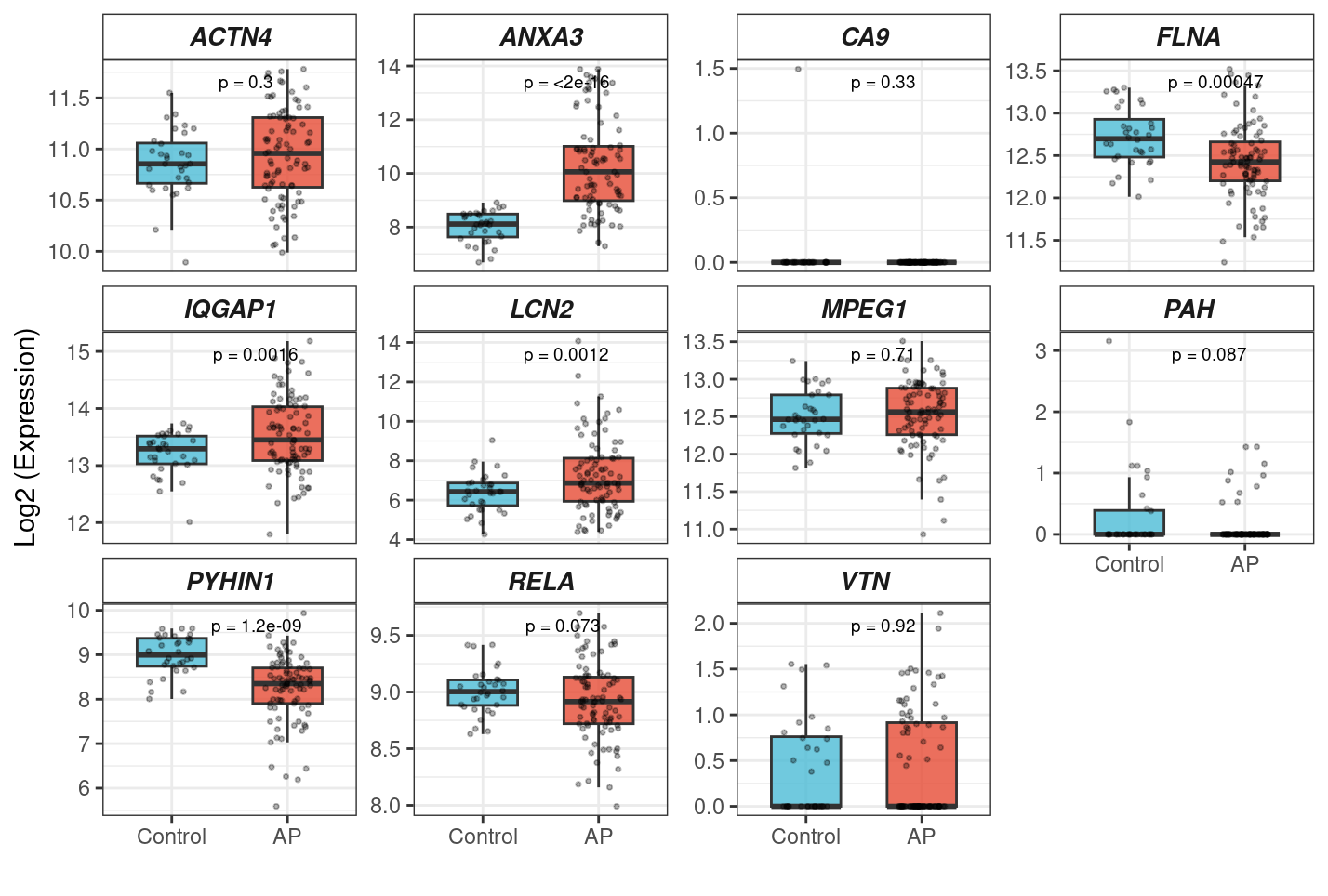

Supplement: S1 Fig — Boxplots illustrating the relative expression levels of 11 pyroptosis-related differentially expressed genes (PRDEGs)—RELA, IQGAP1, ACTN4, FLNA, ANXA3, VTN, LCN2, MPEG1, PAH, CA9 (homologous to murine Car9), and PYHIN1—were generated within the independent human peripheral blood dataset. This analysis assesses the cross-species translatability of the initial murine-derived signature by comparing the differential expression patterns of these candidate genes between human patients with AP and healthy controls. (TIF) [file pone.0347204.s001.tif]

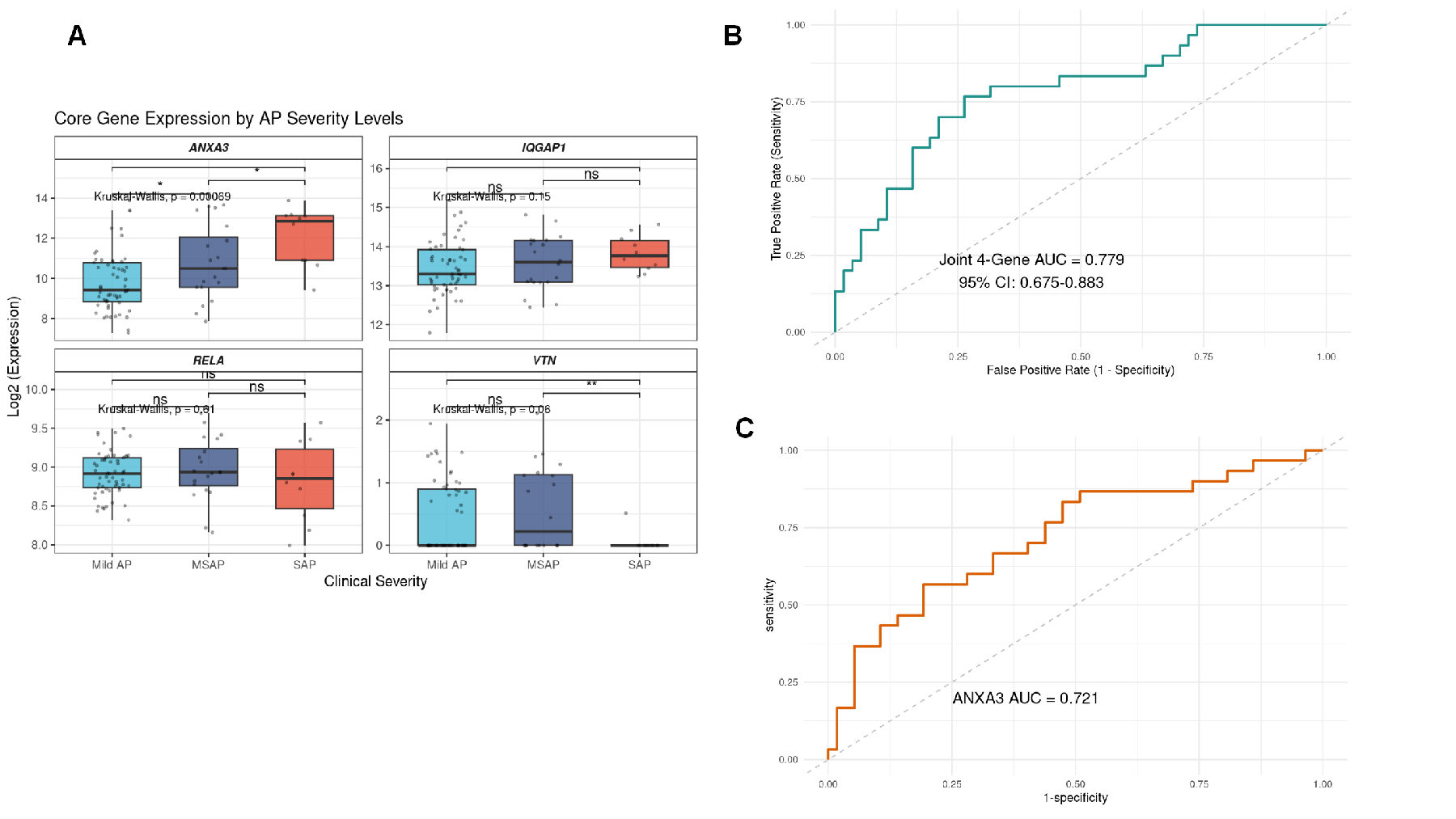

Supplement: S2 Fig — The figure depicts the relationship between gene expression and the severity of acute pancreatitis (Mild, Moderate, and Severe) using a human peripheral blood dataset. (A) Boxplots show the relative expression levels of the signature genes across the three severity groups, emphasizing significant differential expression as the disease progresses. (B-C) ROC curves assess the discriminatory ability (AUC) of the multi-gene panel compared to ANXA3 alone in predicting disease severity. (TIFF) [file pone.0347204.s002.tiff]

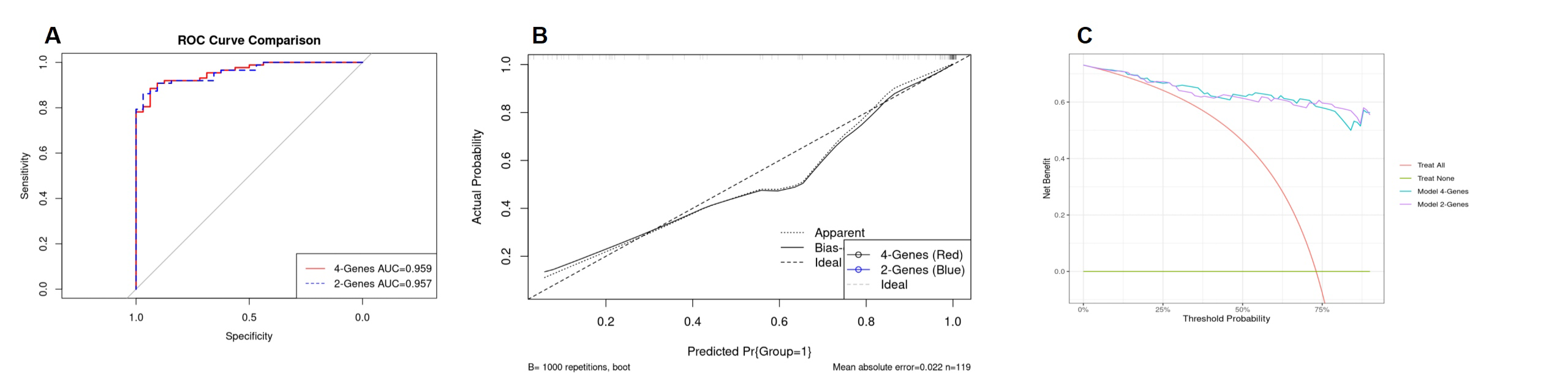

Supplement: S3 Fig — The figure illustrates the diagnostic performance, calibration, and clinical utility of the initial four-gene signature (ANXA3, IQGAP1, RELA, and VTN) compared to the AIC-optimized two-gene signature (ANXA3 and IQGAP1) in the human validation cohort. Performance is evaluated through ROC curves to assess discriminatory accuracy (AUC) (A), calibration plots (B) to evaluate the goodness-of-fit between predicted and observed outcomes, and (DCA) (C) to determine clinical net benefit across various threshold probabilities. (TIF) [file pone.0347204.s003.tif]

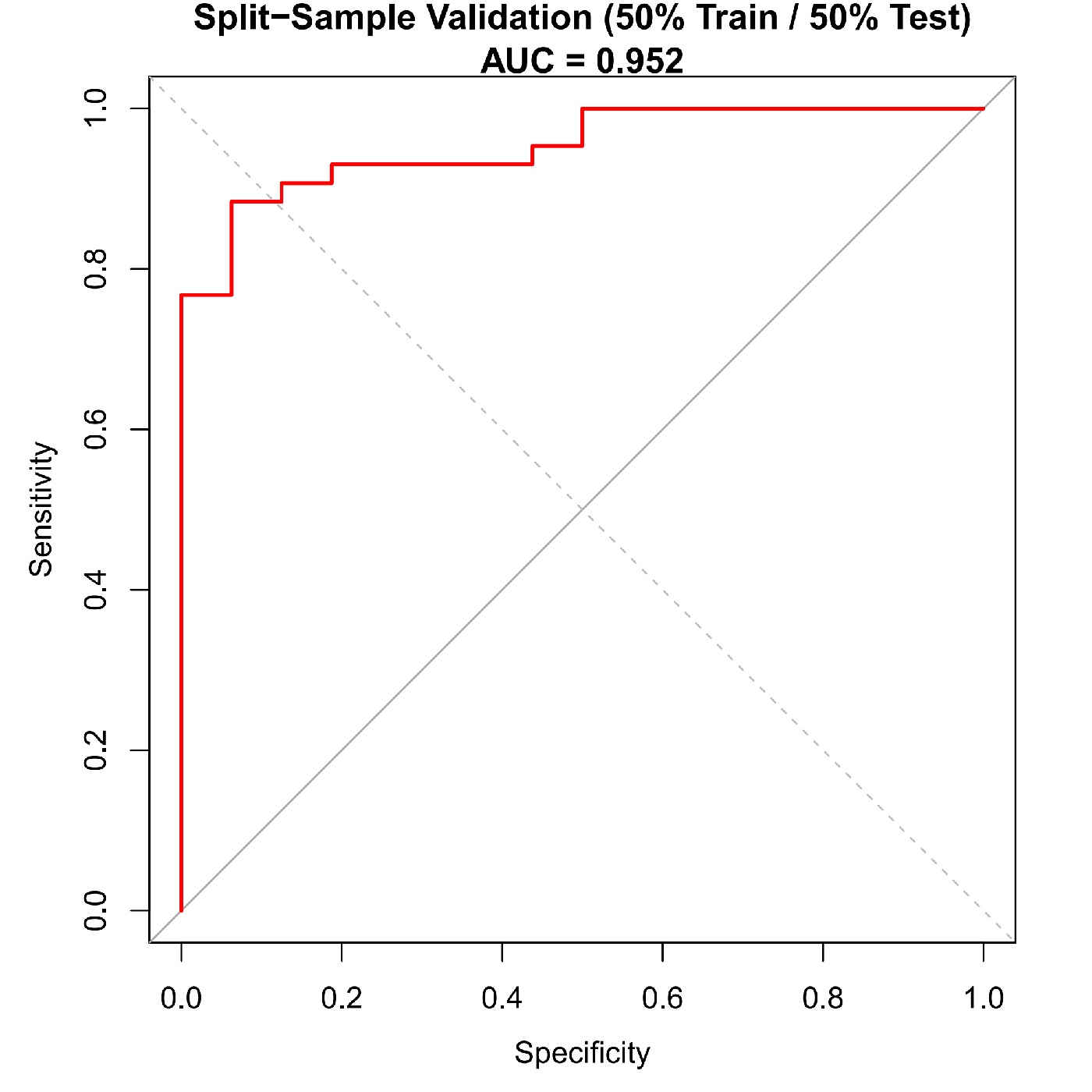

Supplement: S4 Fig — The ROC curve was used to assess the discriminatory accuracy of the optimized two-gene signature (ANXA3 and IQGAP1) in an independent human testing set. To mitigate the risk of overfitting, the external human cohort (GSE194331, N = 119) was randomly divided into a training set (50%, n = 60) for coefficient re-estimation and an independent testing set (50%, n = 59). The curve illustrates the model’s performance when applied exclusively to the testing set, demonstrating that the high diagnostic accuracy is robust and not a result of overfitting. (TIFF) [file pone.0347204.s004.tiff]
